# The importance of simulated lung fluid (SLF) extractions for a more relevant evaluation of the oxidative potential of particulate matter

**DOI:** 10.1038/s41598-017-11979-3

**Published:** 2017-09-14

**Authors:** Aude Calas, Gaëlle Uzu, Jean M. F. Martins, Didier Voisin, Lorenzo Spadini, Thomas Lacroix, Jean-Luc Jaffrezo

**Affiliations:** Univ. Grenoble Alpes, CNRS, IRD, IGE, Grenoble, F-38 000 France

## Abstract

Particulate matter (PM) induces oxidative stress *in vivo*, leading to adverse health effects. Oxidative potential (OP) of PM is increasingly studied as a relevant metric for health impact (instead of PM mass concentration) as much of the ambient particle mass do not contribute to PM toxicity. Several assays have been developed to quantify PM oxidative potential and a widely used one is the acellular dithiothreitol (DTT) assay. However in such assays, particles are usually extracted with methanol or Milli-Q water which is unrepresentative of physiological conditions. For this purpose, OP_DTT_ measurements after simulated lung fluids (SLF) extraction, in order to look at the impact of simulated lung fluid constituents, were compared to Milli-Q water extraction measurements. Our major finding is a significant decrease of the OP_DTT_ when the artificial lysosomal fluid (ALF) solution was used. Indeed, ligand compounds are present in the SLF solutions and some induce a decrease of the OP when compared to water extraction. Our results suggest that the effect of ligands and complexation in lining fluids towards PM contaminants probably has been underestimated and should be investigated further.

## Introduction

Since the late 80′s, epidemiologic studies have shown associations between ambient particulate matter (PM) and adverse health outcomes^1, 2^. It is now known that airborne particulate matter can induce lung, cardiovascular and cerebrovascular diseases^3^. A crucial process that causes the adverse health effects of PM appears to be oxidative stress involving both acellular oxidant-generating characteristics of the particles (shape, size, solubility, surface reactivity, chemical composition, etc.) and cellular properties including the PM ability to stimulate cellular oxidant generation^4^. Cellular reactive oxygen species (ROS) can be produced by several mechanisms and ROS production can be catalytically enhanced by redox active chemical species (organic chemicals and metals) encountered, absorbed in, or adsorbed on ambient PM^4–6^. In other words, PM health effects can be attributed to the oxidative potential (OP) of ambient particles, PM can provide ROS or induce their production in the airways, and if the amount exceeds available antioxidant defenses, an oxidative stress ensues^7^. This oxidative stress shoulders many physiological and pathophysiological processes that are relevant to public health.

Much of the ambient particle mass consists of low toxicity components such as ammonium sulfates/nitrates, sea salt, and crustal dust^8^. While these contribute substantially to the mass, they do not contribute to PM toxicity. Conversely, trace species such as transition metals and some organics contribute little to mass but can be major contributors to PM toxicity^8^. This has led to the hypothesis that PM’s oxidative potential is a key parameter in understanding health impacts and is a better predictor of PM toxicity than PM mass concentration^9, 10^ or even partial chemical composition^11^. OP integrates the effect of size, surface properties, and chemical composition of PM, to give a unique metric^12^. As a result, in the last decade assays have been developed to quantify the oxidative potential of whole ambient aerosols or of constituent chemical components^13–16^. These include *in vitro* cellular assays (macrophages and epithelial cells), *in vivo* assays (quantification of markers of airway/systemic oxidative stress and inflammation) and *in vitro* acellular assays (DTT, AA, ESR, DCFH etc.)^6, 17–23^. All these assays are somewhat specific and no real consensus has emerged towards a standard test^8, 24^. However, among all these tests, acellular assays, besides being non-invasive, have the advantages of being fast, easy to organize, inexpensive as compared to cellular tests, and they can be automated.

Acellular assays include tests based on chemical (depletion of antioxidant, titration)^6, 21, 22^ or physical measurements (ESR)^23^. A widely used measure of PM oxidative potential is the dithiothreitol (DTT) assay^16^, which was developed to simulate the *in vivo* generation of superoxide radicals (0_2_^−^). DTT is indeed commonly used as a surrogate for biological reducing agents (NADH and NADPH)^16, 25^ or as GSH simulant^26^. OP measurements from this assay are commonly correlated with markers of airway inflammation^20, 27^ or of oxidative stress (HO-1)^19^. Moreover, some epidemiological studies have shown close associations between DTT assay and ED (emergency department) visits for wheezing and congestive heart failure^28^ or, prevalence of asthma symptoms and rhinitis^28, 29^.

DTT assay is both used to assess the OP of environmental PM samples and/or the OP of model chemical compounds. This assay is known to be sensitive to some compounds (Cu, quinones, etc.) but the whole OP_DTT_ observed in ambient samples may be affected from chemical compounds not sufficiently taken into account. Investigating the link between OP_DTT_, aerosols chemical compounds and speciation is necessary to better link specific compounds or aerosol sources to the observed health outcomes^30^.

In studies on PM or chemical species oxidative potential, particles are usually extracted with methanol or Milli-Q water^9, 25^. The use of methanol aims at maximizing the amount of reactive compounds extracted from PM by increasing their solubility. However methanol, as well as Milli-Q water, differ in various properties (such as pH, ionic strength, absence of complexing ligands etc.) from physiological fluids encountered by PM in lung^30^.

This is important, as recent toxicological studies suggest that compounds both soluble and insoluble in lung fluids can cause inflammation but the reaction may be faster for soluble compounds (immediately internalized). A direct measurement of the bioavailable fraction is not easy, and measurements of lung bioaccessibility have been developed as a surrogate, providing an estimation of the bioavailable fraction of reactive compounds^31^. Lung bioaccessibility is defined as the amount of a substance soluble in a simulated lung fluid^32^. Metal bioaccessibility in the lung has been studied for the last 15 years^30^. But to our knowledge, there is no available study on organic compounds bioaccessibility in lung, and no proposition for a standardized analytical method as yet. This results in wide variability among reported extraction methods for bioaccessibility measurement: the type of leaching agents used, pH, temperature, or extraction methods (shaking, centrifugation, sonication etc.). However, despite this variability across different studies, the bioaccessibility of some metals seems to be enhanced when using extraction solutions that more closely mimic the physiological conditions^30, 32–34^.

In this paper, we report not on the measurement of bioaccessibility but on DTT assay modified design using extraction solutions that mimic lung fluid composition in order to look at the impact of simulated lung fluid constituents on the OP assays and in order to be closer to real physiological conditions. Three simulated lung fluids (SLF) were tested: the Gamble’s solution, the artificial lysosomal fluid (ALF)’s solution and Gamble’s solution added with dipalmitoylphosphatidylcholine (DPPC) *i.e*. the major phospholipid of lung surfactant. Individual tested compounds (reference materials) were chosen for either, their known toxicity, their role as source markers, their abundance in atmospheric aerosol, or for their known positive response to the DTT assay. Real environmental samples from different origins (ambient PM from filters and certified reference material CRM) were also studied. OP measurements with the DTT assay after SLFs extraction were compared to measurements with Milli-Q water extraction in order to evaluate the impact of this alternative design of the DTT assay.

## Results and Discussion

### Rate of DTT loss for reference materials in the four extracting solutions

The rate of DTT loss (nmol.min^−1^) or DTT depletion was assessed for different concentrations of selected individual compounds. Further, the rate of DTT loss *vs* molar concentration relationship (e.g. the dose-response relationship) was established for each compound and each extracting fluid by calculating the regression equation. Only DTT depletions above the LOD (limit of detection) and DTT depletion by Zn^2+^ were considered. The LOD in each media, was defined as three times the standard deviation (SD) of DTT depletion in media blanks^9^ (detailed information concerning the LOD determination is available in the supplementary information 1, Tables S1 and S2).

**Figure 1:**
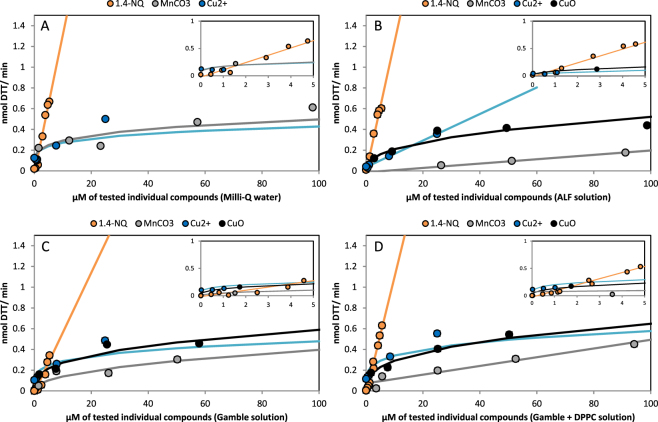
Rate of DTT loss vs molar concentration relationship for 1,4-NQ, MnCO_3_ and Cu^2+^ and CuO in the 4 extraction solutions (**A**) Milli-Q water, (**B**) ALF solution, (**C**) Gamble solution, (**D**) Gamble + DPPC solution (Bars correspond to SD of triplicate).

Some compounds induced very low or slight DTT depletion (regression slope near zero and/or with R^2^ < 0.6) for all the studied media and all tested concentrations. These compounds are benzo(a)pyrene (BaP) and PAH (Polycyclic aromatic hydrocarbon) derivatives: 7 H-benz(de)anthracene-7-one (BA), Benzo(b)naphtho(2,1-d)thiophene (BNT), 3-methylchrysene as well as levoglucosan, NH_4_^+^, Ca^2+^ + SO_4_^2−^ and Zn^2+^  + SO_4_^2−^. Since PAHs are not redox active, they should not induce an oxidation of DTT^13^. However, it is known that PAHs can indirectly induce oxidative stress, through *in vivo* biotransformation by the P450 cytochrome, epoxide hydrolase and the dihydrodial dehydrogenase detoxification pathway^8, 19^. Further, studies assessing the OP of ambient PM have shown strong correlations between DTT losses and PAH contents of the samples. However, these correlations can be attributed to the correlation between PAH and quinones or other organic compounds such as secondary organic aerosol (SOA), which are redox-active^13, 19, 35–38^. In the case of the other non-reactive to DTT compounds, the reaction between the redox couple DTT_ox_/DTT (E = −0.33 V) and the redox couple Ca^2+^/Ca = −2.87 V or Zn^2+^/Zn = −0.76 V is not favored thermodynamically (*i.e*. is redox inert). In the case of CaSO_4_, SO_4_^2−^ was the ion in the solution tested towards the DTT assay, and it showed no reactivity with DTT. Zinc sulfate was also tested and allowed to evaluate Zn^2+^ complexation phenomena by DTT. Conversely, MnCO_3_, Cu^2+^ (+2 Cl^−^), CuO and the quinone 1,4-NQ induced a DTT depletion during the assay. The results are summarized in the Fig. 1 and Tables 1 to 4 (Table 1: chemicals tested in Milli-Q water, Table 2: ALF solution, Table 3: Gamble solution, Table 4: Gamble + DPPC solution).

**Table 1:** Chemicals tested in Milli-Q water.

Chemicals in Milli-Q water	Concentration range (µM)	Regression equation	R²	N	n
Benzo(a)pyrene (BaP)	9.5 × 10^−5^–9.2	y = 0.0090x + 0.0074	0.11	6	18
7H-Benz(de)anthracene-7-one (BA)	3.7 × 10^−5^ – 7.1	y = 0.0021ln(x) + 0.021	0.81	6	18
Benzo(b)naphtho(2,1-d)thiophene (BNT)	1.1 × 10^−4^ – 7.0	y = 0.0014x + 0.0030	0.19	6	18
3-methylchrysene	1.4 × 10^−4^ – 7.7	y = 0.0011x + 0.0067	0.17	6	18
1,4-naphtoquinone	8.6 × 10^−4^ – 5.3	y = 0.13x − 0.026	0.98	10	25
Levoglucosan	1.00 – 101	y = −0.0020ln(x) + 0.019	0.15	5	15
NH_4_^+^	99.8	nc		1	2
SO_4_^2−^	0.522–415	y = 0.000030x + 0.012	0.53	5	15
MnCO_3_	1.55–97.8	y = 0.17x^0.23^	0.71	5	15
Cu^2+^	0.025–25	y = 0.17x^0.20^	0.63	5	15
CuO	1.72–100.4	na			
Zn^2+^	0.025–24.1	y = −0.0021x + 0.0049	0.72	5	15

The table presents the regression equations (rate of DTT loss *vs* molar concentration relationship), the concentration range and the determination coefficients of the tested individual compounds. For each concentration (N) measurements (n) were realized in duplicate or triplicate (example: with BaP, triplicates were realized for each tested concentration, n = 3*N = 3*6 = 18).na no regression found nc not calculated, results < LOD for all concentration range. N number of concentrations used for the regression. n number of measurements

**Table 2:** Chemicals tested in the ALF solution.

Chemicals in ALF solution	Concentration range (µM)	Regression equation	R²	N	n
Benzo(a)pyrene (BaP)	7.7 × 10^−5^ – 9.2	nc		6	18
7H-Benz(de)anthracene-7-one (BA)	5.0 × 10^−5^ – 7.0	nc		6	18
Benzo(b)naphtho(2,1-d)thiophene (BNT)	5.6 × 10^−5^ −7.0	y = −0.00090x + 0.0060	0.13	6	18
3-methylchrysene	2.0 × 10^−4^ – 7.5	nc		6	18
1,4-naphtoquinone	8.3 × 10^−4^ – 5.4	y = 0.13x − 0.015	0.98	8	24
Levoglucosan	0.947–100	nc		5	15
NH_4_^+^	92.6	nc		1	2
SO_4_^2−^	0.620–398	nc		5	15
MnCO_3_	9.43–91.1	y = 0.0021x − 0.014	0.98	4	12
Cu^2+^	0.027–25	y = 0.013x + 0.037	0.99	5	15
CuO	2.8–99	y = 0.085x^0.39^	0.92	5	15
Zn^2+^	0.03–25	y = −0.00020x − 0.0026	0.40	5	15

The table presents the regression equations (rate of DTT loss *vs* molar concentration relationship), the concentration range and the determination coefficients of the tested individual compounds. For each concentration (N) measurements (n) were realized in duplicate or triplicate. nc not calculated, results < LOD for all concentration range. N number of concentrations used for the regression. n number of measurements.

**Table 3:** Chemicals tested in the Gamble solution.

Chemicals in Gamble solution	Concentrations range (µM)	Regression equation	R²	N	n
Benzo(a)pyrene (BaP)	7.7 × 10^−5^ – 9.5	y = −0.0011x + 0.0088	0.56	6	18
7H-Benz(de)anthracene-7-one (BA)	9.7 × 10^−5^ – 6.6	y = 0.0019x + 0.0091	0.25	6	18
Benzo(b)naphtho(2,1-d)thiophene (BNT)	7.2 × 10^−6^ – 6.9	y = − 0.0027x + 0.0067	0.27	6	18
3-methyl chrysene	9.5 × 10^−5^ – 8.1	y = −0.0020ln(x) + 0.0050	0.38	6	18
1,4-naphtoquinone	9.4 × 10^−4^ – 5.4	y = 0.058x – 0.016	0.90	10	25
Levoglucosan	0.990–102	y = 0.0019ln(x) + 0.0045	0.32	5	15
NH_4_^+^	99.6	nc		1	2
SO_4_^2−^	0.495–393	y = 0.0010ln(x) − 0.0068	0.45	5	15
MnCO_3_	1.52–108	y = 0.049x ^0.45^	0.90	5	15
Cu^2+^	0.025–25	y = 0.17x ^0.22^	0.79	5	15
CuO	1.7–58	y = 0.13x ^0.33^	0.92	4	12
Zn^2+^	0.026–25	y = −0.0021x + 0.0098	0.84	5	15

The table presents the regression equations (rate of DTT loss *vs* molar concentration relationship), the concentration range and the determination coefficients of the tested individual compounds. For each concentration (N) measurements (n) were realized in duplicate or triplicate. nc not calculated, results < LOD for all concentration range. N number of concentrations used for the regression. n number of measurements.

**Table 4:** Chemicals tested in Gamble + DPPC solution.

Chemicals in Gamble + DPPC solution	Concentrations range	Regression equation	R²	N	n
Benzo(a)pyrene (BaP)	7.8 × 10^–5^–9.2	y = 0.00090x + 0.017	0.27	6	18
7H-Benz(de)anthracene-7-one (BA)	4.0 × 10^−5^ – 7.1	y = −0.0010x + 0.014	0.22	6	18
Benzo(b)naphtho(2,1-d)thiophene (BNT)	4.18 × 10^−3^ – 6.8	y = 0.0018x − 0.0094	0.29	4	12
3-methylchrysene	2.0 × 10^−4^ – 7.7	y = − 0.00070x + 0.011	0.18	6	18
1,4-naphtoquinone	9.5 × 10^−4^ – 5.6	y = 0.11x – 0.026	0.98	12	36
Levoglucosan	1.02–103	y = 0.000060x – 0.016	0.15	5	15
NH_4_^+^	98.9	nc		1	2
SO_4_^2−^	0.487–403	y = −0.00002x − 0.018	0.62	5	15
MnCO_3_	3.57–94.2	y = 0.0042x + 0.073	0.94	5	15
Cu^2+^	0.024–24.8	y = 0.20x^0.23^	0.83	5	15
CuO	1.7–50	y = 0.13x^0.35^	0.95	4	12
Zn^2+^	0.029–25.3	y = −0.0080ln(x) − 0.0066	0.99	5	15

The table presents the regression equations (rate of DTT loss *vs* molar concentration relationship), the concentration range and the determination coefficients of the tested individual compounds. For each concentration (N) measurements (n) were realized in duplicate or triplicate. nc not calculated, results < LOD for all concentration range. N number of concentrations used for the regression. n number of measurements.

Concerning the extractions in Milli-Q water (Table 1 and Fig. 1A), a linear dose-response relationship was observed for 1.4 NQ and Zn^2+^. A power dose-response relationship was observed for MnCO_3_ and Cu^2+^. Such first and second order reactions have already been observed by Charrier and Anastasio (2012)^13^ with 1,4-NQ, Mn^2+^ and Cu^2+^.

However, while Charrier and Anastasio (2012)^13^ observed higher DTT depletion by Cu^2+^ than by Mn^2+^, in the present work, a quasi-similar DTT loss was observed for Cu^2+^ and MnCO_3_ (y = 0.17x  ^0.20^ and y = 0.17x  ^0.23^, respectively). This difference may be due to the different speciation, Mn^2+^ for Charrier and Anastasio^13^, whereas in this study, an insoluble species of Manganese (MnCO_3_) was used. This may have induced a surface reaction, as already observed in DTT assay^39, 40^, rather than a redox cycling reaction.

Copper oxide induces DTT depletion when extracted with Milli-Q water but, due to the difficulty to maintain CuO in stable suspension in Milli-Q water, no dose-response relationship was observed (no regression found).

For a theoretical concentration of 1 µM, the DTT depletion by 1,4-NQ was lower than those by MnCO_3_ and Cu^2+^. Because of the power law dose-response relationships found for MnCO_3_ and Cu^2+^, this pattern tends to change at higher concentrations, *i.e*. above 2 µM. However, quinones concentrations in ambient air are often lower than trace metal concentrations. Indeed, concentration levels of oxygenated PAHs in the environment are in the pg.m^−3^ to lower ng.m^−3^ range (depending on the compound of interest, sampling period and place)^41–43^ whereas trace metal element make up 1 to 20% of PM mass (depending on size fraction and time of the year)^44, 45^. These results show the importance of the DTT depletion by Cu and Mn and support the results of Charrier and Anastasio (2012)^13^.

Finally, a negative dose-response relationship was observed for Zn^2+^ (Table 1 and Figure S5). The same observation has been made for PbO (Figure S11) measured in a preliminary step of this study.

In the case of the ALF solution (Table 2), which presents lower pH (4.5) and higher citrate content among the different SLF solutions, a dose-response relationship was obtained for 1,4-NQ, Cu^2+^, MnCO_3_, and CuO (regression with R^2^ > 0.6). A linear dose-response relationship was observed for all these compounds with the exception of CuO, for which a power law dose-response relationship was calculated. For 1,4-NQ, MnCO_3_, Cu^2+^ and CuO positive relationships were obtained with DTT depletion (normalized to 1 µM) ranging as follows: 1,4-NQ > CuO > Cu^2+^ > MnCO_3_ (Fig. 1B). For Zn^2+^, negative results were again observed at all tested concentrations (Table 2 and Figure S5).

For the Gamble solution (Table 3), a clear dose-response relationship was established for 1,4-NQ, MnCO_3_, Cu^2+^, Zn^2+^ and CuO (regression calculated with R^2^ > 0.6). A negative linear response was again observed for Zn^2+^ (Figure S5). For CuO, DTT depletion during the experiment was over 70% for the highest CuO concentration (100 µM). Consequently this concentration was not used in the regression, indeed over 70% the catalytic redox reaction rate cannot be simplified as linear^46^. For the same theoretical concentration of 1 µM, DTT depletion ranging as follows: Cu^2+^ > CuO > MnCO_3_ ~1,4-NQ. Except for 1,4-NQ (linear dose-response relationship) these patterns tend to change at higher concentrations because of the power-law dose-response relationships observed for Cu^2+^, CuO and MnCO_3_ (Fig. 1C).

As observed in Milli-Q water and ALF solution, the rates of DTT loss by BaP, BA, BNT, 3-methylchrysene, levoglucosan, NH_4_^+^, and SO_4_^2−^ were insignificant.

Finally, for the Gamble + DPPC solution (Table 4), a positive linear dose-response relationship was observed for 1,4-NQ, MnCO_3_ while a positive power law dose-response relationship was observed for Cu^2+^, CuO.

For the same theoretical concentration of 1 µM, the DTT loss measured in the Gamble + DPPC solution, were similar to those measured in the Gamble solution and ranged in the following order: Cu^2+^ > CuO > MnCO_3_ ~1,4-NQ. A slight evolution can be seen in the highest concentrations due to the observed non-linear dose-response relationships (Fig. 1D). In opposition to the other fluids, in this medium a negative logarithmic dose-response relationship was obtained for Zn^2+^ (Figure S5).

Considering the highest R² values, the best regressions among the extraction solutions were observed in the Gamble + DPPC solution. This is consistent with the findings of Foucaud *et al*. (2007)^47^ who showed an improvement of suspension stability in the presence of DPPC.

### Impact of the SLF extraction towards DTT loss results

We compared the rate of DTT loss in the four extracting solutions for 1,4-NQ, MnCO_3_, Cu^2+^, CuO and Zn^2+^. All these compounds presented a clear dose-response relationship.

For 1,4-NQ, DTT losses were not significantly different in Milli-Q water, ALF or Gamble + DPPC solutions. The following linear relationships were respectively determined: y = 0.13x −0.026, y = 0.13x −0.015 and y = 0.11x −0.026 and the determination coefficients were always higher than 0.9. Conversely, in the Gamble solution, DTT depletion was lower than in the other solutions (y = 0.058x −0.016) (Figure S1).

For MnCO_3_, the highest DTT depletion (normalized at 1 µM), was observed in Milli-Q water (y = 0.17x^0.23^), followed by the Gamble + DPPC (y = 0.0042x + 0.073) and Gamble solutions (y = 0.049x^0.45^), and finally the ALF solution (y = 0.0021x −0.014) (Figure S2).

For CuO, similar DTT depletion were found in the Gamble + DPPC and the Gamble solution (y = 0.13x^0.35^ and y = 0.13x^0.33^, respectively). Similar to MnCO_3_, the rate of DTT loss in the ALF solution was the lowest (y = 0.085x^0.39^) (Figure S3).

For Cu^2+^ normalized at 1 µM, the higher DTT depletion was found in the Gamble + DPPC solution (y = 0.20x^0.23^) then in Milli-Q water and in the Gamble solution which presented equivalent losses (y = 0.17x^0.20^ and y = 0.17x^0.22^). Finally the lowest depletion was observed in the ALF solution (y = 0.013x + 0.037) (Figure S4).

In contrast, DTT loss by Zn^2+^ (y = −0.0002x −0.003) was less negative in the ALF solution than in the Gamble + DPPC, Gamble solutions, and in Milli-Q water (y = −0.0080 ln(x) −0.0066, y = 0.0021x + 0.01 and y = −0.002x + 0.005, respectively) (Figure S5).

The mechanisms that would permit to explain all these differences are not yet fully understood, although some hypotheses can be made:

### DTT depletions are affected by pH

There is no issue of acidity with the DTT assay since a phosphate buffer was used to maintain a constant and physiological pH value. No absorbance differences of blanks were observed between the four solutions (supplementary information 2, Figure S6). Moreover, no differences have been found between DTT depletion in the four media blanks (Figure S7). However, pH can affect the dissolution during the extraction phase of the chemicals tested. Metals carried by PM can undergo increased dissolution when pH is lowered and the ALF solution, with the lowest pH among SLF solutions, is known to allow better dissolution of insoluble compounds such as CuO or ZnO^33, 48^.

### Ligand content and type of reactions (surface/solution reactions, complexation etc.)

Complexing anions and functional groups known to chelate metals are present in the tested SLF solutions: orthophosphates, carbonates, carboxyls (from glycine, citrate) and amines (from glycine). Detailed information concerning the composition of the SLF solutions is available in Table S3. The Gamble and Gamble + DPPC solutions present higher glycine contents than the ALF solution. However, the ALF solution presents the higher citrate content (20 mg.L^−1^) and so, the strongest chelating potential among the tested extracting fluids. The chelating strength of these solutions can be ordered as follows: ALF solution > Gamble + DPPC solution ~Gamble solution. The impact of the added DPPC on the chelating strength has not been studied here, consequently no assumption regarding this point was made.

As an organic compound, 1,4-NQ was not expected to form strong complexes with such species from SLF solutions, and was thus supposed to behave similarly in all fluids. However, in the Gamble solution, 1,4-NQ had a lower response towards DTT than in the other media. It can be assumed that this medium is not as favorable to electron transfer as the other solutions. This highlights the possible role of DPPC in electron transfer since DTT depletions are higher in the Gamble + DPPC solution compared to the Gamble solution. Furthermore, the transformation of 1,4-NQ in solution cannot be ruled out, as 1,4-NQ aging has been observed during storage but also during the extraction phase (Figure S11), thus possibly explaining the weaker DTT depletion by 1,4-NQ in the Gamble solution.

Chemical speciation calculations conducted with the PhreeqC^49^ code showed that Cu^2+^ forms several complexes in the Gamble solution. For a 25 µM concentration of CuCl_2_ in the Gamble solution calculations showed that Cu^2+^ primarily forms complexes with glycine and to a lesser extent with citrate and carbonate. Cu^2+^, the free species, should not account for more than 0.0006%. However, DTT depletion by Cu^2+^ was very similar in Milli-Q water and the Gamble solution, likely because glycine-Cu complexes present the same redox potential as Cu^2+^, as shown by Mohamed *et al*. (2012)^50^. The higher Cu^2+^ reactivity in the Gamble + DPPC solution can be explained by the potential role of DPPC as an electron transfer facilitator. Cu^2+^ speciation in the ALF solution was also calculated with PhreeqC: Cu^2+^ primarily forms citrate complexes, which can explain the observed lowest DTT depletion in the ALF solution as citrate-Cu complexes redox potential is reduced as compared to Cu^2+^ ^51^.

Concerning Zn^2+^, a negative dose-response relationship was observed whatever the extraction media indicating that more DTT was oxidized in blanks than in samples (Table 1 to Table 4 and Figure S5). As already observed previously by Held *et al*. (1996)^52^, this can be explained by a complexation process between DTT and Zn^2+^, which may have stabilized DTT from oxidization^40^. The same assumption can be applied to PbO^39^ (Figure S11). However and compared to the other tested solutions, the ALF solution exhibited different patterns of DTT depletion rate, with fewer negative linear dose-response relationships. It shows that, in this fluid, DTT is less protected by its complexation with Zn^2+^, which preferentially reacts with the other ligands of the ALF solution (citrate etc.). Once again differences were observed between the Gamble and Gamble + DPPC solutions (linear vs logarithmic dose-response relationship).

Rate of DTT loss by the insoluble MnCO_3_ in the tested media decreases (when MnCO_3_ < 50 µM) as the concentration of ligands in the SLF solution increases: Milli-Q water > Gamble solution ~Gamble + DPPC solution > ALF solution. These results can be explained by a minor or absence of constituent effect of the Gamble and Gamble + DPPC solutions on MnCO_3_ dissolution. The main mechanism involved is likely a surface reaction and a surface occupation of reactive sites by SLF constituents. In the case of the ALF solution, which presents the lowest pH, MnCO_3_ could be partially dissolved during the extraction phase and further Mn^2+^ complexation with citrate decreases DTT depletion.

DTT losses by the insoluble CuO were very close in both Gamble and Gamble + DPPC solutions, DTT loss by CuO in the ALF solution was lower than in the other fluids and can probably be explained in the same manner as for MnCO_3_, *i.e*. through a partial dissolution followed by interactions/complexations with citrate. However, this effect is less pronounced than for MnCO_3_ probably because of the even lower solubility of CuO.

In summary, DTT depletion measured within the four extracting solutions varied among the tested compounds and also with their interaction with the chemicals present in solution, which have variable chelating strengths (citrate – glycine - DTT). It should be noted that, citrate was primarily used to replace the proteins of the lung lining fluid^53^ (such as iron or copper-binding proteins), which can act as a local defense system against oxidative reactions^54^. As illustrated with CuO results, it should also be noted that SLF solutions facilitate the OP measurement when compared with Milli-Q water results. To go further, oxidative potential of ambient PM has been measured after extractions in the four media.

### OP_DTT_ results from ambient PM and implications according to the extraction solution

OP_DTT_ is defined as the mass-normalized DTT depletion for a given substance (nmol DTT.min^−1^.µg^−1^). The OP_DTT_ of ambient PM was evaluated for the three SLF solutions and for Milli-Q water to look at the effect of the SLF composition on the OP of complex PM. In France, particulate matter sanitary alert are based on PM_10_ measurement. PM_10_ studied in this paper, were collected on filters by the certified associations for the monitoring of the air quality (AASQA) and came from the urban background of Nice (southeast of France), from the city of Passy (French Alpine Valley) and from a subway station of Toulouse (southwest of France). Two certified reference materials (ERM CZ 120 and NIST 1649 b) were also tested.

The filters tested were chosen randomly in terms of sampling dates and came from various locations. The chemistry involved is thus quite different and non-specifically representative of the collection sites (average concentration and composition). The objective was not to compare the different locations but rather to evaluate the oxidative potential of very different PM_10_ in the three SLF, to start exploring the role of their composition on the PM_10_ toxicity as measured within the DTT assay.

**Figure 2:**
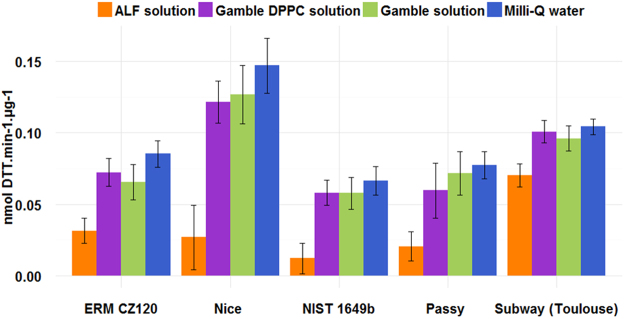
OP_DTT_ of PM from different locations and extracted in the different media (bars correspond to standard deviation of duplicate).

**Figure 3:**
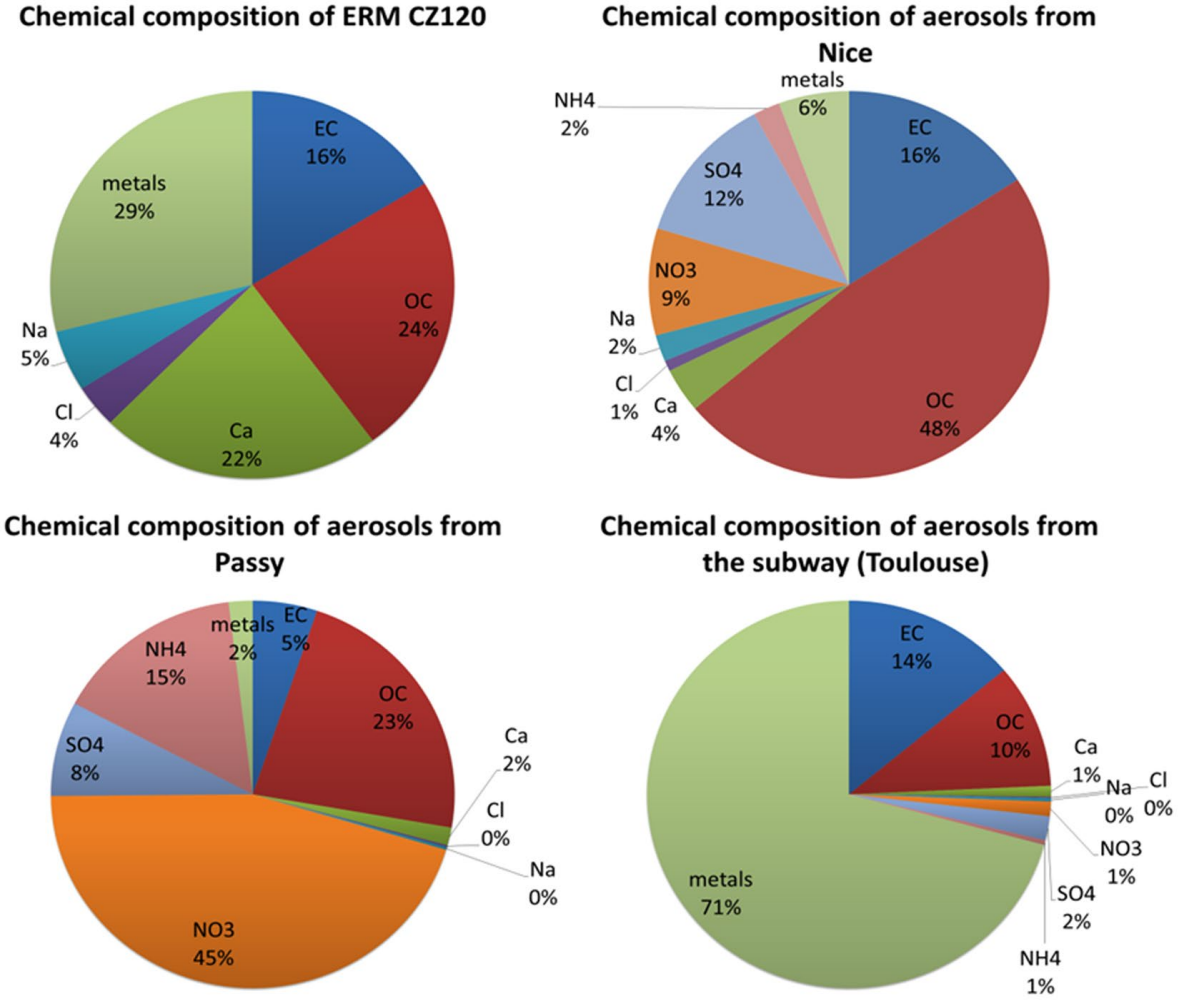
Chemical composition of PM from different locations (mass%).

DTT depletion per cubic meter (nmol DTT.min^−1^.m^−3^) was not calculated as for the CRMs these calculations were impossible. Figure 2 presents the OP_DTT_ of the different tested aerosols (realized in duplicate) in the four model fluids. Figure 3 shows the chemical composition of the main compounds for the three locations and for ERM CZ120 (such complete chemical composition was not available for NIST 1649b).

The OP_DTT_ measured in Milli-Q water, Gamble and Gamble + DPPC solution does not present statistically significant differences. Conversely, the ALF solution led to OP results significantly lower than in the other solutions (Man-Whitney statistical test, p-value < 0.05) (Supplementary information 3, Table S4 and Figure S8). However, it has to be noted that the results obtained in Milli-Q water were systematically higher than in the other extraction solutions.

The samples present a different chemical composition (Fig. 3) which can provide some clarifications about the previous OP results.

The overall lower OP results obtained in the ALF solution can be linked to citrate, a strong metal chelator present in this solution. PM from Toulouse’s subway presented the lowest OP variations among all SLF solutions and the highest DTT depletion observed in the ALF solution. Dissolution is increased in the ALF solution due to the low pH during extraction (see bioaccessibility percentage for ERM CZ 120 in the Table S7) and this is combined with a strong complexation of citrate leading to the poor available of metal for DTT reaction. However, high OP results from subway PM after ALF extraction could have been triggered by a greater concentration of free metallic ions than for other PM (highest content of metals, 71%) due to the low pH of ALF solution and further less complexed by citrate since high concentrations of dissolved species were available. It could also be assigned to metal surface reaction^39^ due to high metal-rich content partially insoluble (mainly oxides). With 29% metal content, the ERM CZ120 sample also presented a noticeable DTT depletion after ALF extraction supporting the previous hypotheses. Moreover, to end with discussion regards ALF extraction, result for the PM from Nice whose composition is driven by high OC, presents a strong diminution of OP compared to other extractions solutions. High content of OC that usually correlates with high OP values which, presently, do not drive anymore OP results when accounting for ALF extraction. Such result can also add in favor the importance of metals when using ALF extractions solution. In the case of Passy, the lower OP were observed among all locations, the results can be explained by the high nitrate content (45% wt) which is non-reactive to DTT and by the lowest metal content observed among the different aerosols (2%). Finally, for Nice, significantly higher OPs for all extraction solutions (except ALF) were observed and may highlight the importance of organic compounds (48% in such samples), such as SOA, in the OP of ambient PM^19, 35–38^.

However, the result for the PM from Nice after the ALF solution extraction, which presents a strong diminution of the OP, cannot rule out an inhibition of the DTT oxidation by the organic compounds in presence of ALF ligands (since organic reference compounds presented also < LOD OP results after extraction). Studies on the OP of environmental samples often use metal chelator agents as DTPA in order to inhibit metal ion-induced ROS^55, 56^. However, Charrier and Anastasio (2012)^13^ showed that the addition of EDTA suppresses DTT response from metals and surprisingly, also from quinones. But, no other metal chelating agents (DTPA, citrate etc.) have been studied. This result may support our observation of an inhibition of DTT depletion by organic compounds in presence of chelating agents.

In this study, DTT assay has been applied in conditions more closely mimicking lung physiology. Interactions between the lung fluids constituents and OP have been observed. The study using reference compounds highlighted the importance and role of specific interactions between chemical species (Cu^2+^…), their speciation, and SLF organic ligands (such as glycine or citrate): they were shown to be able to decrease the OP of these elements through mechanisms involving chelation and/or surface reactions. The decrease or increase of OP values for reference compounds after SLF extraction was unpredictable compared to Milli-Q water extraction. This could be a significant source of biases in correlations with health outcomes studies that could be addressed by further improvements in OP assays. Our results with the CuO also suggest that the organic constituents of the SLF solution allowed a better suspension and as a consequence facilitate measurement of the OP within the DTT assay.

The ALF solution simulates inflammatory conditions as may be encountered during PM pollution peaks. Studying OP of PM_10_ with DTT assay in this particular media has shown interesting results. Extractions in the ALF solution induce a significant difference in comparison with others. OP results with the DTT assay is significantly lower in this media and can be explained by complexation phenomena (mainly with citrate). Then, no significant differences have emerged between Milli-Q water, Gamble or Gamble + DPPC solution extractions. However, a constant pattern is observed with always higher OP values in Milli-Q water than in the other extraction solutions. This trend cannot rule out a possible statistically significant difference on a more important number of samples (here, only 5 samples) or in other environments, as such differences exist among reference compounds. However, all these observations have been made for the DTT assay and cannot be generalized to other acellular assays.

This alternative protocol using extractions in SLF solutions before OP measurement allow to focus on PM compounds really available for ROS generation. All these results suggest that the effect of ligands and complexation in lining fluids probably has been underestimated and should be investigated further. Moreover, our results indicate that combining information from OP measurements and PM chemical composition could be important for understanding the complex mechanisms that can bias OP measurements. OP measurements through the DTT assay appeared correlated with some health outcomes^28, 29^, other OP measurements were not always good indicators of PM toxicity^28, 29, 57, 58^. Such differences could be assigned to difference into assay designs. In the future, results of such new design, with SLF extraction before OP measurement, could be compared to epidemiological data in order to evaluate if it can improve the links between OP measurement and observed pathologies. Finally, OP assays standardization is compulsory to go forward, so as to allow epidemiology-OP measurement cross-studies.

### Methodology

#### Reagents

Potassium dihydrogen phosphate, di-potassium hydrogen phosphate, magnesium chloride, sodium chloride, potassium chloride, sodium hydrogen phosphate, sodium sulfate, calcium chloride dihydrate, sodium acetate, sodium hydrogen carbonate, sodium citrate dihydrate, sodium hydroxide, citric acid, sodium tartrate dihydrate, sodium lactate, sodium pyruvate, dithiothreitol and 5,5′-dithiobis-(2-nitrobenzoic acid) were obtained from Roth Sochiel EURL (Lauterbourg, France). 1,2-dipalmitoylphosphatidylcholine (DPPC) was obtained from Avanti. 1,4-Naphtoquinone, Manganese(II) carbonate, Copper (II) chloride, Copper(II) oxide, Zinc sulfate heptahydrate, 7 H-benz(de)anthracene-7-one, Benzo(b)naphtho(2,1-d)thiophene, 3-methylchrysene and levoglucosan were obtained from Sigma-Aldrich (France). Sulfate ion and ammonium ion were obtained from Analab (France). Benzo(a)pyrene was obtained from Alfa-Aesar.

#### Samples

PM sampling: Ambient particles were collected by filtration during 24 h (24 × 30 m^3^.h^−1^) with DIGITEL DA-80 (high volume samplers) on quartz filters (Tissuquartz Pallflex). The filters were calcined at 500 °C for 8 h before use. After sampling, the filters were folded, wrapped in aluminum foils sealed in polyethylene bags and stored at −15 °C until analysis^44^. Samples from different locations with diverse compositions were used in this study: from Passy known to present a large contribution of domestic biomass burning to the PM mass^59^, ambient PM from the urban background of Nice with significant marine contribution to the PM mass, and ambient PM collected at a subway station (Toulouse) with high metal content. ERM CZ 120 and NIST 1649 b were used as certified reference material (CRM).

Chemicals species from PM: Six PAH and PAH derivatives (Benzo(a)pyrene (BaP), 7H-Benz(de)anthracene-7-one (BA), Benzo(b)naphtho(2,1-d)thiophene (BNT(2,1-d), 3-methylchrysene, 1,4 naphtoquinone (1,4-NQ), one common anhydro sugar (levoglucosan), two ions (NH_4_^+^ and SO_4_^2−^), and four metals (Manganese (II) carbonate, Copper (II) chloride, Copper (II) oxide, Zinc sulfate heptahydrate) were tested.

Different concentrations of soluble or insoluble compounds have also been tested for different speciation (CuCl_2_ and CuO). As much as possible, the tested concentrations were in the range of atmospheric concentrations^42, 59–61^ taking into account the average volume of air inhaled by an adult (around 10 m^3^.day^−1^) and the average volume of lung fluid (20 ml)^32^. All these conditions, as well as justification of compounds selected, are summarized in Table S5.

### Preparation of DTT, DTNB, phosphate buffer and positive control

Solutions of DTT and DTNB were prepared in phosphate buffer (1 M, pH of 7.4 ± 0.1) (at 0.5 mM and 1 mM, respectively) and maintained in ice and in the dark during the experiment. The phosphate buffer was treated with Chelex® 100 sodium form resin to remove any metal contamination.

The stock solution for positive control consisted of 1,4 naphtoquinone solubilized in methanol (986 ± 1 µM). Intermediate solutions of 1,4-NQ of 24.5 ± 0.5 µM were prepared in a phosphate buffer before each experimental day in order to get 2 µM of the compounds in the reaction volume.

### Simulated lung fluid (SLF)

Three simulated lung fluids (SLF) have been tested. These fluids were designed to model the interactions of PM with extracellular lung fluids^53^. The Gamble’s solution represents the interstitial fluid deep within lungs and is mixture of salts^53^ (pH: 7.4). Lung macrophages are one of the main producers of ROS in the early inflammation phase. To compensate ROS production, and possibly to modulate ROS release, macrophages use antioxidant defenses^62, 63^. ALF’s solution mimics fluid after phagocytosis by alveolar and interstitial macrophages (pH: 4.5) and simulate inflammatory conditions^64, 65^. The third SLF is the Gamble’s solution supplemented with dipalmitoylphosphatidylcholine (DPPC) (pH: 7.4). This major phospholipid of lung surfactant helps reducing surface tension at the air-water interface of the terminal airways and improves lungs defensive function^66^. The citrate in the SLF solutions replaces proteins that can be found in lung lining fluid and acetate was used instead of organic acids^53, 64^.

### PM suspension

Filter punches of 0.196 cm² or individual compounds were placed in 15 mL tubes for extraction. The extraction consisted in 2 h of vortex agitation at 37 °C for the filters, CRM and the insoluble reference compounds. Soluble compounds were vortexed 40 minutes at 37 °C. Extraction time and procedure were optimized and can be found in the supplementary information 4, Figures S9, S10 and S11.

For the ambient PM and CRM study, the PM mass tested (1 punch in 2 ml of extraction solution) are 0.49 µg, 1.01 µg, 0.68 µg respectively for Nice, Toulouse and Passy and 0.86 µg for the two CRM (supplementary information on the filters is available in supporting information 6).

### DTT assay

Experimentation for DTT depletion induced by reference materials was conducted in 48 well plates whereas the experimentation for the DTT depletion induced by ambient PM was conducted in 96 well plates.

This test consisted of monitoring DTT depletion when in contact with PM; the rate loss of DTT (when in excess) is proportional to the concentration of redox-active species present in PM^9, 39^. A semi-automated procedure was used for the DTT assay with a plate-reader TECAN spectrophotometer Infinite® M 200 pro, and 48 and 96 well CELLSTAR® multiwall plates from Greiner bio-one®. DTT depletion was monitored for 30 minutes with the following procedure: first, measurement of the intrinsic absorbance (Abs_int_) of particles and substrate at 412 nm was made. Then, for experiments conducted in the 48 well plates, 25 nmol of DTT (50 µL of 0.5 mM DTT solution in phosphate buffer) was injected, then for each sample (blank included) DTT was quantified immediately (t = 0) and after 15 and 30 minutes (t = 15 and t = 30) of exposure, with samples in triplicate (100 nmol of 5,5′-dithiobis(2-nitrobenzoic acid) – 100 µL of 1 mM DTNB in phosphate buffer). Because of the high repeatability between the triplicates (average CV = 3.2%) (Supplementary information 5, Table S6), positive controls (50 µL of 24.5 ± 0.5 µM 1,4-NQ solution) were quantified only once at each measurement time. For triplicate samples, if the coefficient of variation (CV) was higher than 5% the extreme value between the triplicates was removed.

The rate of DTT loss (nmol.min^−1^) was determined from the slope of the linear regression of calculated nmol of consumed DTT *vs* time. The amount of remaining DTT was calculated as follows:

1 $${n}_{DTT,i}=\frac{Ab{s}_{i}\ast {n}_{DTT,0}}{Ab{s}_{t0}}$$

where n_DTT, i_ is the amount of DTT (nmol) at t = i, Abs_i_ is the absorbance at t = i, n_DTT,O_ is the initial amount of DTT (nmol) and Abs_t0_ is the absorbance at t = 0.

The quality of the linear regression was estimated with the coefficient of regression R². The linear regression was considered acceptable when R² > 0.90 and when less than 70% of the initial amount of DTT had been oxidized^46^. The repeatability of measurement was estimated using the positive control (1,4-NQ) and was estimated below a 5% difference (Supplementary information 5, Figures S12 and S13). The intrinsic absorbance was subtracted from the final absorbance and the DTT loss in the blanks (oxidation of 100 µM DTT by dissolved oxygen and no added redox-species) was subtracted from the DTT loss of the samples in order to obtain the actual DTT depletion of the samples.

Mass-normalized DTT depletion rates were also calculated and referred to as OP (nmol DTT.min^−1^.µg^−1^), by dividing the DTT depletion of the sample by the PM mass (µg) added in the wells.

### Statistical analyses

All statistical analyses were carried out using the R statistical software 3.4.0. Non-parametric Mann-Whitney tests were used in order to look at the statistical significance between the different extraction solutions and for the environmental samples. This non-parametric test was chosen because of the small size of the dataset.

## Electronic supplementary material

Supplementary information
