# Supplementary material for: The importance of simulated lung fluid (SLF) extractions for a more relevant evaluation of the oxidative potential of particulate matter
Source: Sci Rep. 2017 Sep 14;7:11617. doi: 10.1038/s41598-017-11979-3 (PMC5599505; doi:10.1038/s41598-017-11979-3)
Supplement: Supplementary file 1 — Supplementary information [file 41598_2017_11979_MOESM1_ESM.doc]

**Supplementary information for the manuscript:** “The importance of simulated lung fluid (SLF) extractions for a more relevant evaluation of the oxidative potential of particulate matter”.

Aude Calas, Gaëlle Uzu, Jean M.F. Martins, Didier Voisin, Lorenzo Spadini, Thomas Lacroix and Jean-Luc Jaffrezo.

Univ. Grenoble Alpes, CNRS, IRD, IGE, Grenoble, F-38 000, France.

**S1. Limit of detection (LOD)**

The limit of detection (LOD) in each media (SLF’s solution and Milli-Q water) was calculated for 48 well plate experiments. It was defined as three times the standard deviation of DTT depletion in media blanks^1^. Table S 1 summaries such LOD obtained for the individual tested compounds (for all the media), but also for ambient PM (Milli-Q water and Gamble + DPPC solution only, arising from experiments realized in our team). As expected, LOD values were function of the SLF solution, with LOD ranging as follows: LOD _Gamble solution_ < LOD _Milli-Q water_ ~ LOD _Gamble_ _+ DPPC solution_ < LOD _ALF solution_ (respectively 0.031, 0.040, 0.041 and 0.088 nmol DTT.min^-1^). The LOD comparisons between individual compounds and ambient PM, and for the Gamble + DPPC solution, shown no significant difference (0.040 nmol.min^-1^ and 0.037 nmol.min-1 respectively). On the other hand, difference between individual compounds (Milli-Q water) and Ambient PM (Milli-Q water) was observed (0.041 and 0.0180 nmol.min-1 respectively). However, this difference can be explained by the low number of observations realized for LOD ambient PM (milli-Q water). LODs for the ambient PM (experiment conducted on 96 well plates) were not evaluated here, because of the limited number of experiment realized (one blank realized by location or CRM and by solution of extraction). However, other experiments realized in our team (Ambient PM – 96 well plates) shown that the LOD was improved by more than 3 times for extractions realized in Gamble + DPPC solution (0.037 nmol DTT.min-1 to 0.012 nmol DTT.min-1 with N=39) and might be explained by the decrease of the volumes and the gas/liquid interface. We made the supposition that this improvement was valid for the other solutions (ALF, Gamble solution and Milli-Q water). Since few differences were found between the LOD derived from the individual compounds blanks and filter blanks (ambient PM) in the Gamble + DPPC solution, the LODs obtained from the individual compounds and for the other extraction solutions were used for calculation of the missing LODs. Table S 2 presents the LODs derived from experiments and calculated LODs (in bold).

**S2. Feasibility of DTT assay in SLF’s**

Using 48 well plates, absorbance scan between 300 and 500 nm (
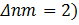
 were realized for the three SLF solutions (ALF solution, Gamble and Gamble + DPPC solution) and for Milli-Q water. 500µL of phosphate buffer and 50µL of SLF solution or Milli-Q water were added in the plate to react with 50µL of DTT at 0.5mM and 100µL of DTNB at 1mM. It should be noted that the reaction product between DTT and DTNB (i.e TNB) is stable for 2 hours^2^ and our measurements were realized immediately after the reaction. No differences, as illustrated by Figure S 6 were observed between the four media with a maximum absorbance of 0.6 at 412nm.

**S3. Statistical analyses**

Figure S 8 and Table S 4 summarize the statistical results of the Mann-Whitney test.

**S4. Extraction optimization (Time and procedure)**

Extraction time efficiency was optimized using ambient PM (2 samples – collected on filter), and reference compounds (PbO and 1.4 NQ). Two extraction solutions were used for the optimization: Milli-Q water and the Gamble + DPPC solution. Three extraction techniques were tested: sonication, vortex at 37°C (vortex in a heat chamber) and a combined extraction (30 minutes of sonication follow by 3 hours of vortex at 37°C). An extraction kinetic was realized. To achieve these, an aliquot was sampled after 1, 30 or 40, 75, 120 and 210 minutes followed by the DTT assay. Two punches of 0.196cm² of ambient samples, PbO and 1.4NQ have been placed in a falcon tube (15ml) with Milli Q water or Gamble + DPPC solution bringing the final concentration to 49µg/ml (sample 1) and 38µg/ml (sample 2) for the ambient PM, 31µM for PbO and 1.4NQ. Blanks (Milli-Q water and Gamble+DPPC solution) have also been realized in order to make sure that no ROS were produced during the extraction step.

Figure S 9 illustrates the results for ambient PM in Gamble + DPPC solution regarding the three extractions techniques. Two different samples were used (the sample 1 for the combined extraction and the sample 2 for the other two extractions). For comparison, the results were normalized per µg of PM (rate of DTT loss per µg of PM) and no differences were observed. However, the vortex method was chosen as it is easier than the combined method and a physiological temperature can easily be maintained in comparison with the sonication.

Concerning the duration of the extraction for the ambient PM, a plateau was reached after 1h15 (76min) of vortex (solution of extraction: Milli-Q water and Gamble + DPPC solution) as illustrated by the Figure S 10. In these experiments the same sample was used (sample 2). For such sample (ambient PM), the time of extraction was fixed over 1h15, i.e. 2h.

The results of the extractions for PbO and 1.4-NQ are presented in the Figure S 11. Regarding PbO, only the first point (1 minute of extraction) shown DTT depletion. Along assay, DTT depletion was
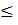
0, i.e. the blank’s depletion was more important than consumption in sample containing PbO. This behavior has already been studied elsewhere and can be explained by a complexation between the DTT and the PbO which prevent the DTT from oxidation ^3–6^. The positive depletion at 1 minute might be explained by the presence of ROS adsorbed at the surface of the lead compound. For the 1,4NQ, a maximum of consumption of DTT was observed at 40 minutes following by a diminution of the depletion rate. This result pointed out the impact of the vortex plus heating on the aging process of 1,4NQ and a potential change of speciation leading to different reactivity.

PM extraction in Milli-Q water before DTT assay are usually range from 30 min to 16h hours ^1,7^. Extraction times, in lung bioaccessibility experiment, range from 15 minutes to few months^8^. Accordingly, our extraction time was set at two hours for insoluble compounds (same as ambient PM) and 40 minutes for soluble compounds, as a seemingly good compromise.

**S5. Repeatability**

Intra-plate as well as inter-plate repeatability was calculated using positive control (1,4-NQ). As a reminder, the stock solution of positive control was solubilized in methanol (986 ± 1 µM). Intermediate solutions of 1,4-NQ of 24.5 ± 0.5 µM were prepared in a phosphate buffer before each experimental day in order to get 2 µM of the compounds in the reaction volume.

Intra-plate repeatability:

The coefficient of variation (CV% = standard deviation/mean*100) of 1.4NQ was calculated between measurements (absorbance) made in triplicates for each dosing time (0, 15 and 30 minutes). Table S 6 presents the CV% obtained for the four media tested (SLF solutions and Milli-Q water) and for different concentrations. The repeatability presents an average coefficient of variation of 3.2% (N=75). As a consequence, DTT depletion by the positive control on the other experiments was quantified only once at each dosing time.

Inter-plate repeatability:

On the basis of the previous conclusion, the inter-plate repeatability was estimated for two consecutive experimental days. 8 measurements of DTT depletion by the positive control were realized (2*4 experiments with intermediate 1.4-NQ solutions from the same stock solution). Figure S 12 presents DTT depletion by the positive control (nmol DTT.min^-1^). The coefficient of variation was estimated at 4.6% (N=8). The repeatability was also evaluated over all experiments achieved for a month. Figure S 13 shows the evolution of the daily average DTT depletion by the positive control over a month of experiments (N=34, January 2016). The DTT depletion ranged between 0.44 and 0.56 nmol.min^-1^ leading to a CV of 8.7%. More precisely a linear increase with a coefficient of determination of 0.89 was obtained if the results from the second day experiment were excluded. During the months of experiments a visual evolution of the 1.4-NQ color was observed, whether for the SLF’s solution or for the positive control realized in methanol. We suspect an evolution of 1.4-NQ speciation in solution (aging). As a result, if the aim is to look at the repeatability, the stock solution of positive control has to be prepared every week (CV <5 %) however if the aim is to look at the quality of the linear regression of the DTT depletion, the stock solution can be used over a longer time.

**S6. Filters from Nice, Toulouse‘s subway and Passy:**

Filters collection date is 24^th^ November 2014, 04^th^ October 2012 and 15^th^ March 2014 respectively for Nice, Toulouse’s subway and Passy. All OP analyses were conducted during April 2016. Other experiments realized in February 2017 did not shown modification of the OP of PM from Nice (DTT assay - results not shown).

**SI References**

1. Fang, T. *et al.* A semi-automated system for quantifying the oxidative potential of ambient particles in aqueous extracts using the dithiothreitol ( DTT ) assay : results from the Southeastern Center for Air Pollution and Epidemiology ( SCAPE ). *Atmos. Meas. Tech.* **8,** 471–482 (2015).

2. Li, Q., Wyatt, A. & Kamens, R. M. Oxidant generation and toxicity enhancement of aged-diesel exhaust. *Atmos. Environ.* **43,** 1037–1042 (2009).

3. Uzu, G. *et al.* In vitro assessment of the pulmonary toxicity and gastric availability of lead-rich particles from a lead recycling plant. *Environ. Sci. Technol.* **45,** 7888–7895 (2011).

4. Goix, S. *et al.* Environmental and health impacts of fine and ultra fine metallic particles : Assessment of threat scores. *Environ. Res.* **133,** 185–194 (2014).

5. Nicolas, J. *et al.* Redox activity and chemical interactions of metal oxide nano- and micro- particles with dithiothreitol (DTT). *Environ. Sci. Process. Impacts.* **17,** 1952–1958 (2015).

6. Krężel, A. *et al.* Coordination of heavy metals by dithiothreitol, a commonly used thiol group protectant. *J. Inorg. Biochem.* **84,** 77–88 (2001).

7. Delfino, R. J. *et al.* Airway inflammation and oxidative potential of air pollutant particles in a pediatric asthma panel. *J. Expo. Sci. Environ. Epidemiol.* **23,** 466–473 (2014).

8. Wiseman, C. L. S. Analytical methods for assessing metal bioaccessibility in airborne particulate matter : A scoping review. *Anal. Chim. Acta.* **877,** 9–18 (2015).

9. Delgado-saborit, J. M., Alam, M. S., Godri-Pollit, K. J., Stark, C. & Harrison, R. M. Analysis of atmospheric concentrations of quinones and polycyclic aromatic hydrocarbons in vapour and particulate phases. *Atmos. Environ.* **77,** 974–982 (2013).

10. Di Filippo, P., Pomata, D., Riccardi, C., Buiarelli, F. & Gallo, V. Oxygenated polycyclic aromatic hydrocarbons in size-segregated urban aerosol. *J. Aerosol Sci.* **87,** 126–134 (2015).

11. Golly, B., Brulfert, G., Berlioux, G., Jaffrezo, J.-L. & Besombes, J.-L. Large chemical characterisation of PM emitted from graphite material production: Application in source apportionment. *Sci. Total Environ.* **538,** 634–643 (2015).

12. Chevrier, F. Chauffage au bois et qualité de llance et rénovation du parc des appareils anciens. (2016).


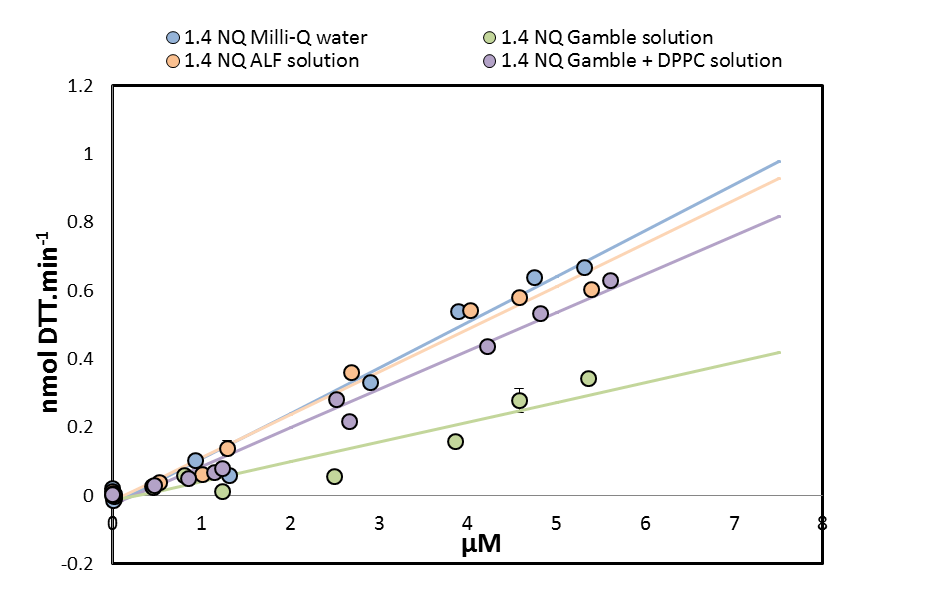


**Figure S 1:** Rate of DTT loss vs molar concentration relationship for 1,4-NQ in the four extraction solutions. (Bars correspond to SD of triplicate).


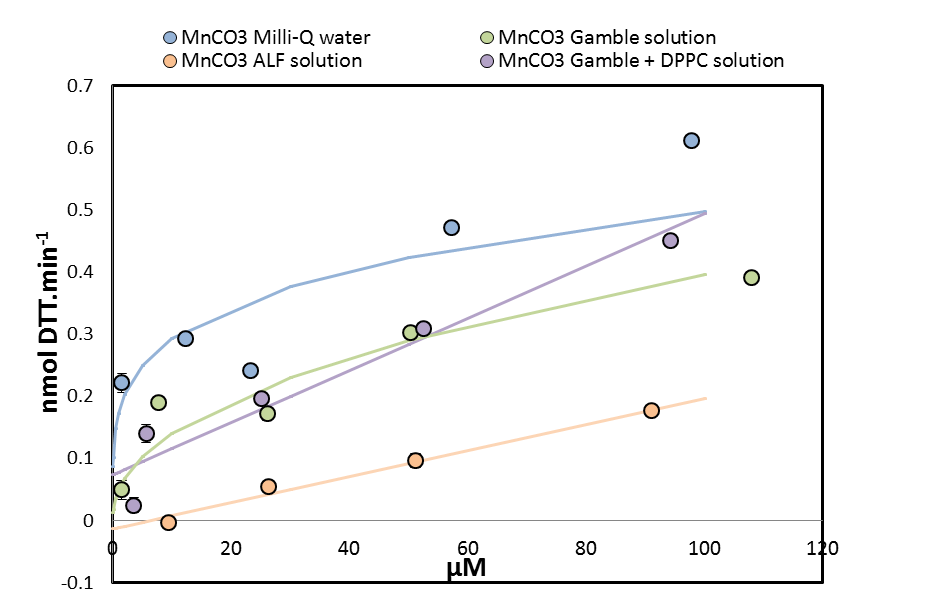
 **Figure S 2:** Rate of DTT loss vs molar concentration relationship for MnCO_3_ in the four extraction solutions. (Bars correspond to SD of triplicate).


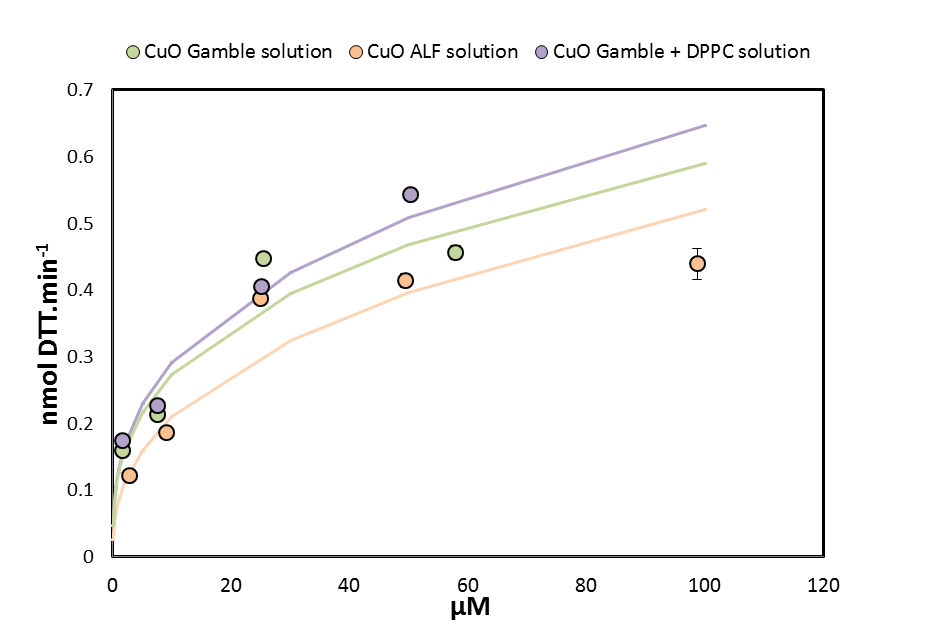
**Figure S 3:** Rate of DTT loss vs molar concentration relationship for CuO in the 3 SLF. (Bars correspond to SD of triplicate).


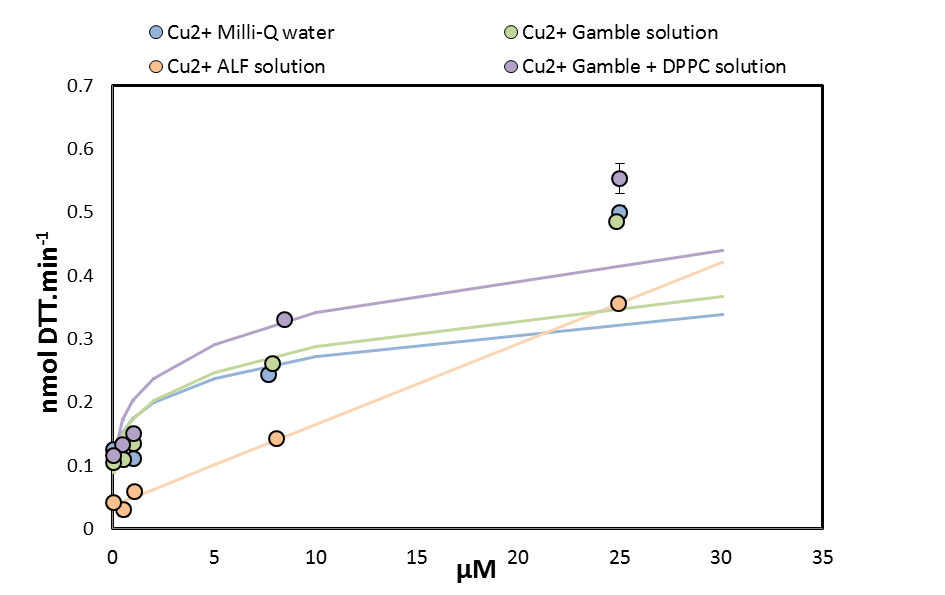
 **Figure S 4:** Rate of DTT loss vs molar concentration relationship for Cu^2+^ in the four extraction solutions. (Bars correspond to SD of triplicate).


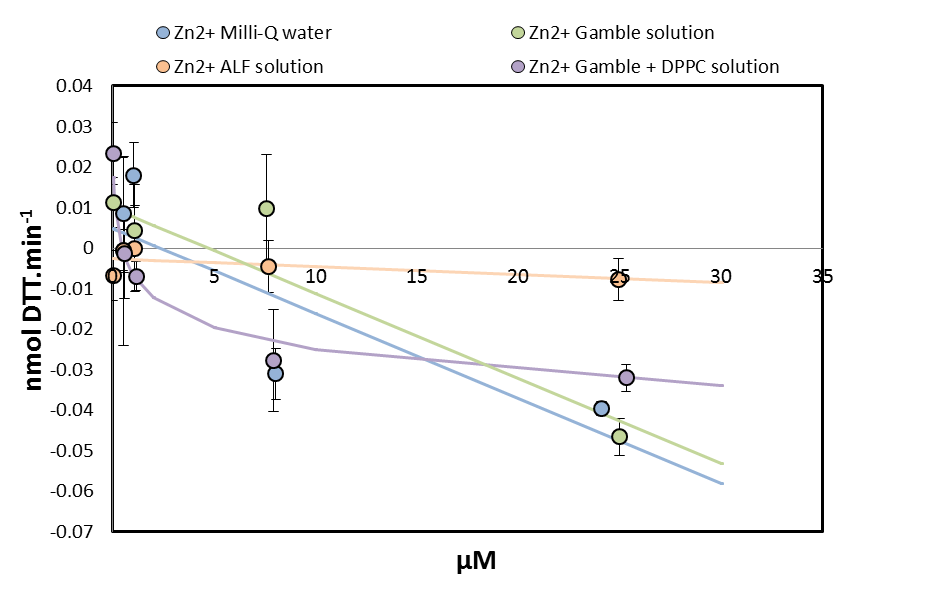
**Figure S 5:** Rate of DTT loss vs molar concentration relationship for Zn^2+^ in the four extraction solutions. (Bars correspond to SD of triplicate).


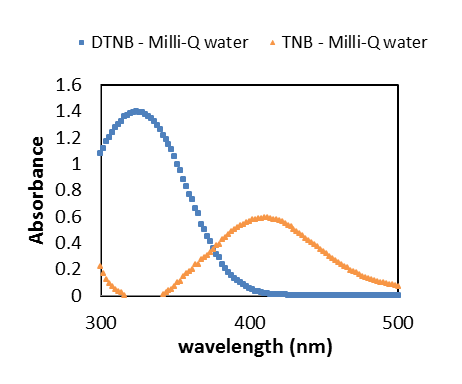

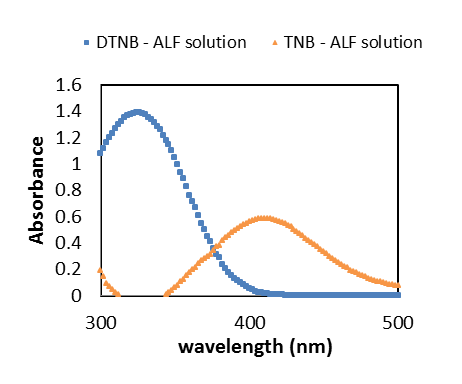

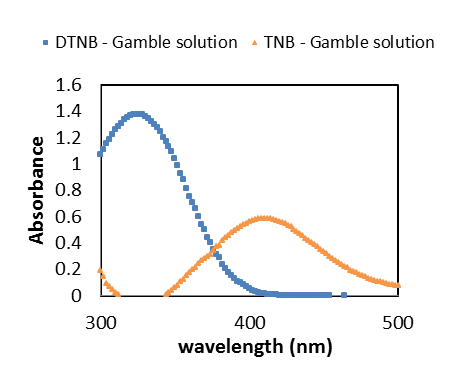

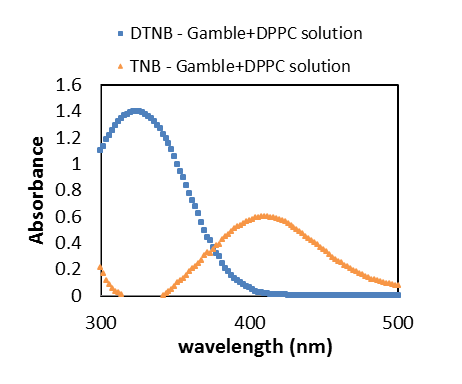
**Figure S 6:** absorption spectra in the 4 extraction solutions between 300 and 500nm. Spectra were realized using a plate-reader TECAN spectrophotometer Infinite® M200 pro. TNB (2-nitro-5-thiobenzoic acid) is determined in the 4 experiments by its absorption at 412nm without any absorption decrease.


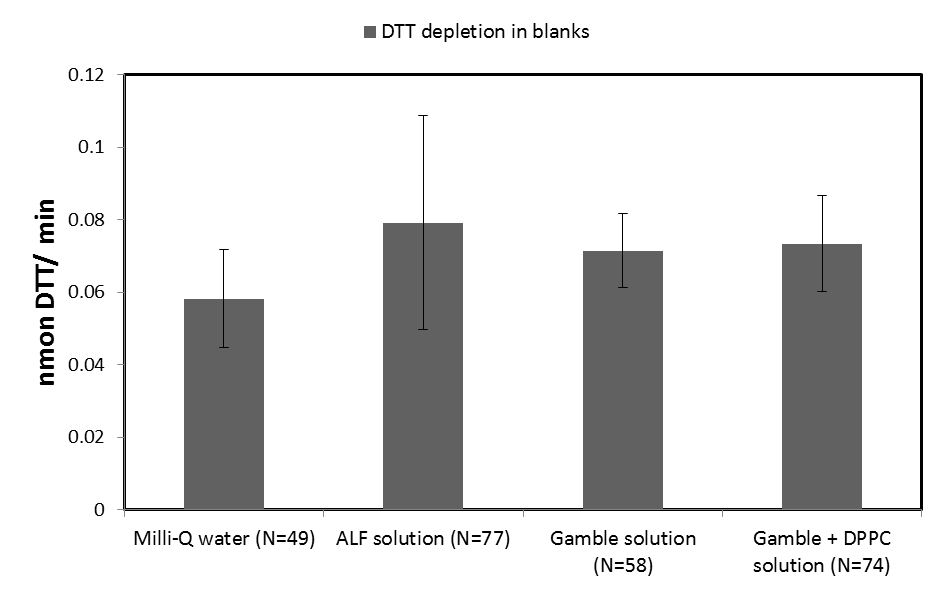


**Figure S 7:** DTT depletion in blanks in the 4 media of extraction (individual compounds study).


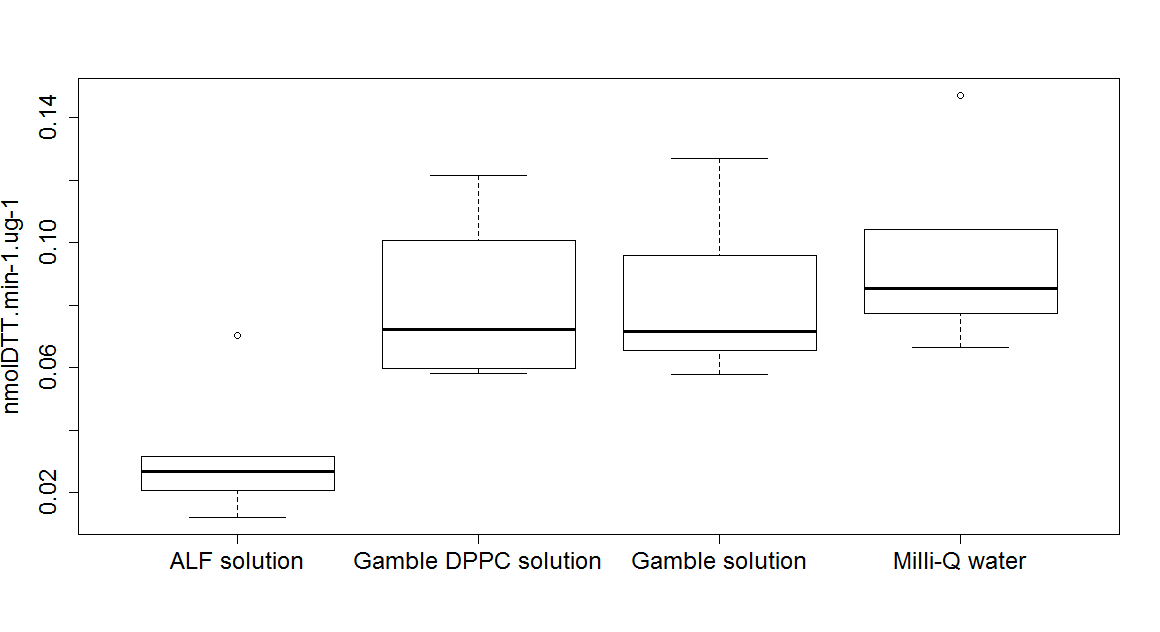


**Figure S 8:** Boxplot of OP DTT obtained from the 4 media of extraction. The ALF solution led to OP results significantly lower than in the other solutions (p-value < 0.05).


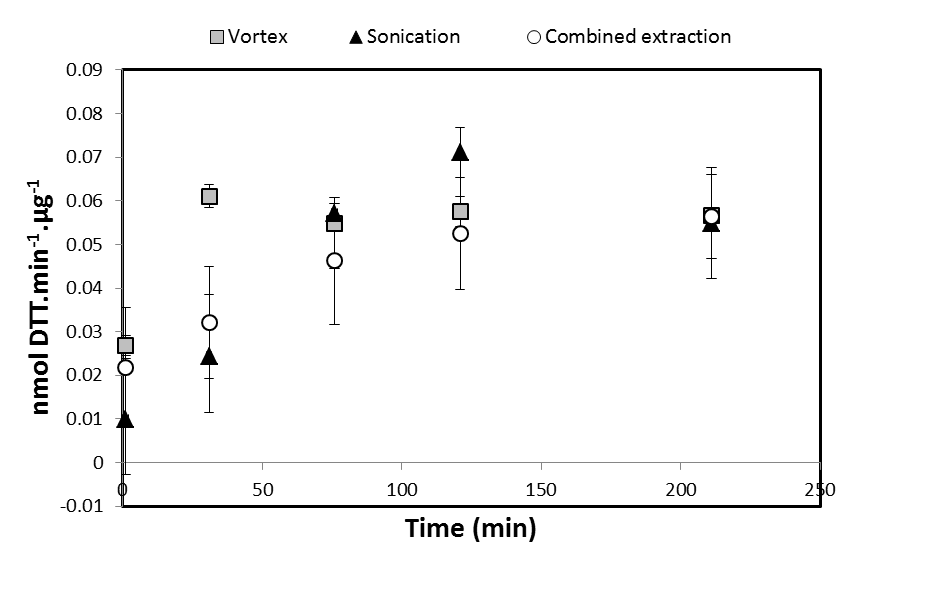
**Figure S 9:** Kinetics of DTT depletion (normalized per µg of PM) when comparing three technics of extractions: vortex, sonication and a combined extraction (sonication followed by vortex). (Bars correspond to standard deviation (SD) of triplicate).


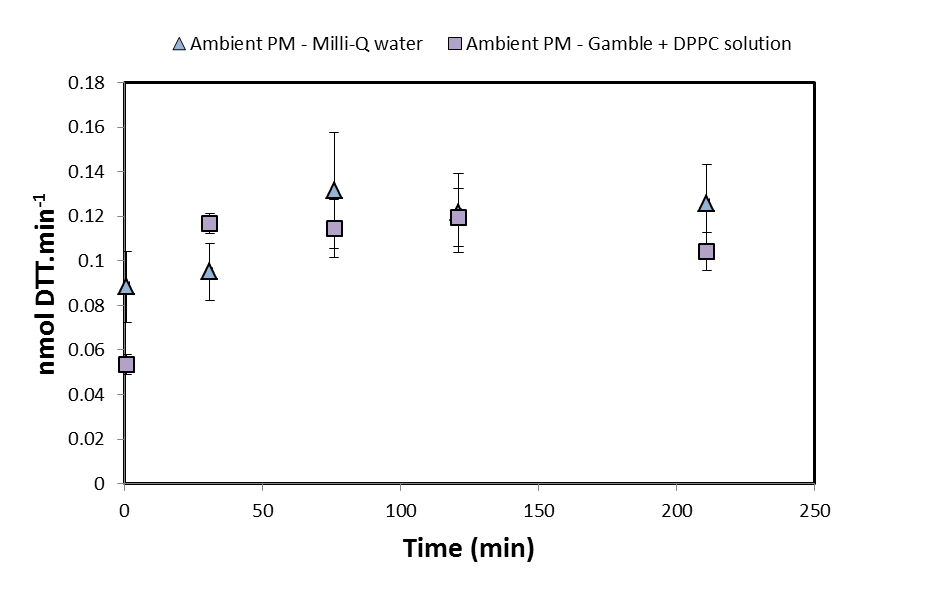
**Figure S 10:** Kinetics of DTT depletion when comparing the same sample extracted in Milli-Q water or Gamble + DPPC solution. (Bars correspond to SD of triplicate).


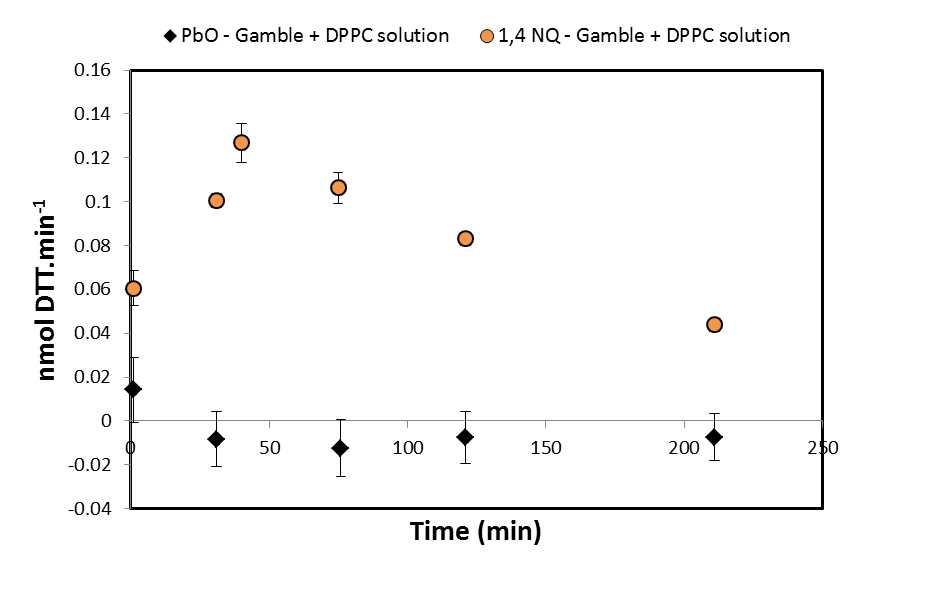
 **Figure S 11:** Kinetics of DTT depletion for individual compounds (PbO and 1,4 NQ) extracted in the Gamble + DPPC solution. (Bars correspond to SD of triplicate).


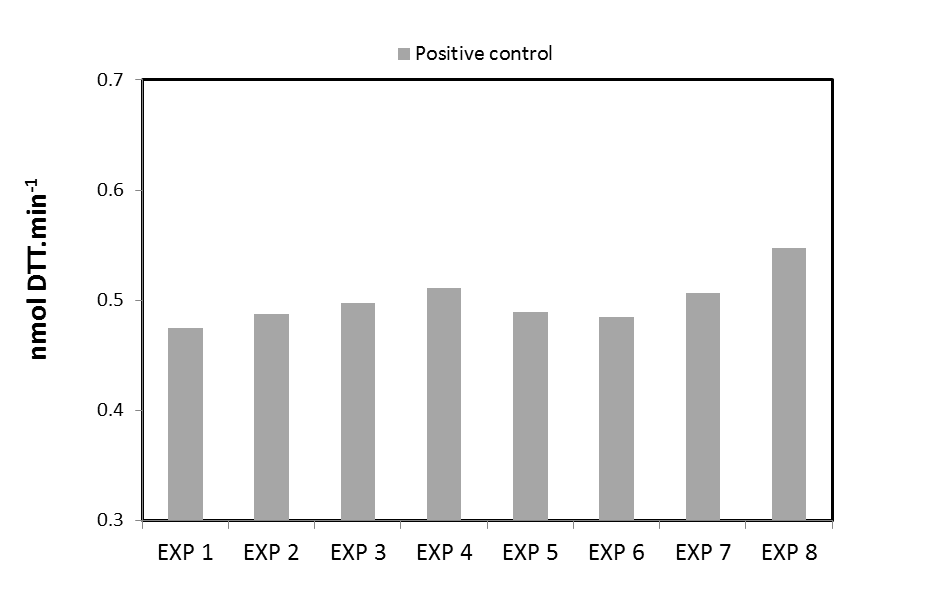
 **Figure S 12:** inter-plate repeatability. DTT depletion by the positive control (1.NQ at 2µM).


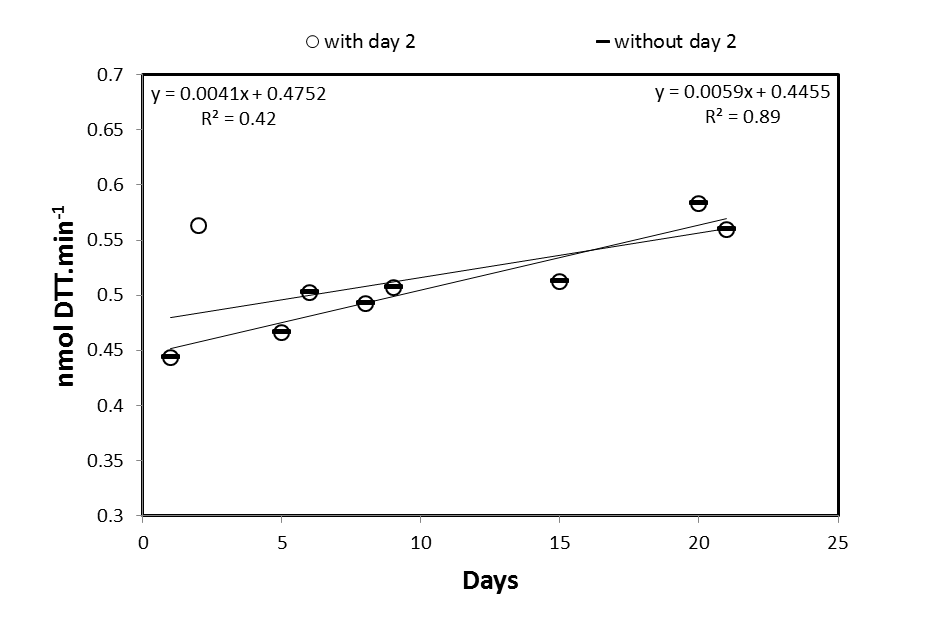
 **Figure S13:** inter-plate repeatability. DTT depletion by the positive control (1.NQ at 2µM).

**Table S 1:** LOD in function of the experimentation (Individual compounds or ambient PM samples) and the extraction solutions – In 48 wells plate. The results for the ambient PM in 48wells arise from experiments realized in our team.

|  | **LOD (nmol DTT.min^-1^)** | **N (observations)** |
| --- | --- | --- |
| **Individual compounds (Milli-Q water)** | 0.041 | 49 |
| **Individual compounds (ALF solution)** | 0.088 | 77 |
| **Individual compounds (Gamble solution)** | 0.031 | 58 |
| **Individual compounds (Gamble + DPPC solution)**  **Ambient PM (Milli-Q water)**  **Ambient PM (Gamble + DPPC solution)** | 0.040  0.018  0.037 | 74  9  42 |

Table S 2: Derived from experiments and supposed (in bold) LODs for 96 well plates.

|  | **LOD – 48 well plates** | **LOD – 96 well plates** |
| --- | --- | --- |
| **Milli-Q water** | 0.041 | **0.012** |
| **ALF solution** | 0.088 | **0.026** |
| **Gamble solution** | 0.031 | **0.0093** |
| **Gamble + DPPC solution** | 0.040 | 0.012 |

Table S 3: Detailed composition of the SLF solutions. The chemical compounds are listed in the order of their introduction to the solution to avoid salt precipitation.

| **Compounds** | **ALF solution** | **Gamble solution** | **Gamble + DPPC solution** |
| --- | --- | --- | --- |
|  | **mg.L^-1^** | | |
| **MgCl_2_** | 50 | 95 | 95 |
| **NaCl** | 3 210 | 6 019 | 6 019 |
| **KCl** |  | 298 | 298 |
| **Na_2_HPO_4_** | 71 | 126 | 126 |
| **Na_2_SO_4_** | 39 | 63 | 63 |
| **CaCl_2_ , 2H_2_O** | 128 | 368 | 368 |
| **CH₃COONa** |  | 574 | 574 |
| **NaHCO_3_** |  | 2 604 | 2 604 |
| **Na_3_ citrate**, **2H_2_O** | 77 | 97 | 97 |
| **NaOH** | 6 000 |  |  |
| **Citric acid** | 20 800 |  |  |
| **Glycine** | 59 | 190 | 190 |
| **Na_2_C_4_H_4_O_6_·2H_2_O** | 90 |  |  |
| **NaC₃H₅O₃** | 85 |  |  |
| **C_3_H_3_NaO_3_** | 86 |  |  |
| **DPPC** |  |  | 0.02% (200mg.L-1) |
| **pH** | 4.5±0.1 | 7.4±0.1 | 7.4±0.1 |

Table S 4: Statistical significance between the different extraction solutions (ambient PM). p-value of the Mann-Whitney statistical test.

Table S 5: Detailed experimental conditions of all selected individual compounds.

| **Compounds** | **Theoretical concentration range (µM)** | **Literature**  **(references)** | **Justification for selection** |
| --- | --- | --- | --- |
| **BaP** | [ 7.5, 1 , 0.5 , 0.01 , 0.001 , 0.0001 ] | 9 | Reglemented |
| **BA** | [ 7.5, 1 , 0.5 , 0.01 , 0.001 , 0.0001 ] | 10 | oxy-PAH - quite abundant |
| **BNT ( 2,1-d )** | [ 7.5, 1 , 0.5 , 0.01 , 0.001 , 0.0001 ] | 11 | derivatives S PAH - specific marker (industry) |
| **3 methylChrys** | [ 7.5, 1 , 0.5 , 0.01 , 0.001 , 0.0001 ] | 12 | derivatives CH3 PAH |
| **1,4 NQ** | [ 5, 4 , 3 , 2 , 1 , 0.5 , 0.025 ,0.01, 0.001 ] | 9 | quinone -known to be reactive in DTT assay |
| **Levoglucosan** | [ 100, 50 , 25 , 7.5 , 1 ] | 12 | specific marker (biomass burning) |
| **NH_4_^+^** | [ 100] | 12 | abundant ion |
| **SO_4_^2-^** | [ 400, 200 , 100 , 1 , 0.5 ] | 12 | abundant ion |
| **MnCO_3_** | [ 100 , 50, 25 , 7.5, 1 ] | Mn total^12^ | insoluble - non-linear response with DTT assay |
| **Cu^2+^** | [ 25 , 7.5 , 1 , 0.5 , 0.025 ] | Cu total^12^ | soluble Copper - non-linear - speciation |
| **CuO** | [ 100 , 50 , 25 , 7.5 , 1 ] | Cu total^12^ | insoluble Copper - non-linear - speciation |
| **Zn^2+^** | [ 25 , 7.5 , 1 , 0.5 , 0.025 ] | Total Zn ^12^ | soluble abundant compound (Zn) |

Table S 6: CV% of the absorbance obtained for each dosing time and for triplicate measurement. 1.4NQ with different concentration and solubilized in the four media was used.

| **CV% of triplicates for each dosing time** | | | |
| --- | --- | --- | --- |
| **Solution of extraction and concentration** | **t=0** | **t=15** | **t=30** |
| **MQ water 1µM** | 3.0 | 3.6 | 3.1 |
| **MQ water 0.5µM** | 2.7 | 2.4 | 2.7 |
| **MQ water 0.025 µM** | 4.3 | 4.2 | 4.6 |
| **MQ water 0.01 µM** | 3.2 | 3.4 | 2.8 |
| **MQ water 0.001µM** | 3.0 | 3.9 | 3.3 |
| **ALF 1µM** | 2.2 | 2.6 | 2.5 |
| **ALF 0.5µM** | 2.1 | 2.4 | 2.2 |
| **ALF 0.025 µM** | 2.6 | 2.4 | 2.2 |
| **ALF 0.01 µM** | 2.5 | 2.5 | 2.2 |
| **ALF 0.001µM** | 2.3 | 2.4 | 2.2 |
| **ALF 5.4 µM** | 2.2 | 5.3 | 4.5 |
| **ALF 4.6 µM** | 2.6 | 3.9 | 5.3 |
| **ALF 4.0µM** | 3.0 | 2.6 | 7.3 |
| **ALF 2.7 µM** | 2.5 | 2.6 | 3.8 |
| **ALF 1.3µM** | 2.4 | 3.0 | 4.0 |
| **Gamble 1µM** | 3.1 | 3.4 | 3.1 |
| **Gamble 0.5µM** | 2.6 | 3.6 | 2.7 |
| **Gamble 0.025 µM** | 4.1 | 4.3 | 4.4 |
| **Gamble 0.01 µM** | 3.1 | 3.3 | 3.1 |
| **Gamble 0.001µM** | 3.1 | 4.0 | 3.1 |
| **Gamble+DPPC 1µM** | 2.6 | 3.2 | 2.8 |
| **Gamble+DPPC 0.5µM** | 4.2 | 4.5 | 4.0 |
| **Gamble+DPPC 0.025 µM** | 3.4 | 3.7 | 2.8 |
| **Gamble+DPPC 0.01 µM** | 3.6 | 3.6 | 3.2 |
| **Gamble+DPPC 0.001µM** | 2.7 | 2.6 | 2.7 |
| **N (observation)** |  |  | **75** |
| **min** |  |  | **2.1** |
| **max** |  |  | **7.3** |
| **average** |  |  | **3.2** |

Table S 7: Bioaccessibility percentage for ERM CZ 120: 24h of extraction in SLF and Milli Q water at 37°C , for 10 mg of ERM-CZ120

|  | **Bioaccessibility (as % of the total metal content)** | | | |
| --- | --- | --- | --- | --- |
| **Element** | Gamble DPPC | ALF | Gamble | Water |
| **Al** | 0 | 1 | 0 | 23 |
| **As** | 65 | 107 | 39 | 103 |
| **Cd** | 80 | 31 | 7 | <5 |
| **Ni** | 5 | 22 | 0 | 1 |
| **Pb** | 2 | 15 | 0 | 1 |
| **Sb** | 1 | 10 | 0 | 0 |
| **Zn** | 2 | 11 | 2 | 0 |
